# Impact of Agricultural Activities on Climate Change: A Review of Greenhouse Gas Emission Patterns in Field Crop Systems

**DOI:** 10.3390/plants13162285

**Published:** 2024-08-17

**Authors:** Yingying Xing, Xiukang Wang

**Affiliations:** Key Laboratory of Applied Ecology of Loess Plateau, College of Life Science, Yan’an University, Yan’an 716000, China; xingyingying@yau.edu.cn

**Keywords:** field crops, environmental impact, soil management, sustainable agriculture, emission reduction strategies

## Abstract

This review paper synthesizes the current understanding of greenhouse gas (GHG) emissions from field cropping systems. It examines the key factors influencing GHG emissions, including crop type, management practices, and soil conditions. The review highlights the variability in GHG emissions across different cropping systems. Conventional tillage systems generally emit higher levels of carbon dioxide (CO_2_) and nitrous oxide (N_2_O) than no-till or reduced tillage systems. Crop rotation, cover cropping, and residue management can significantly reduce GHG emissions by improving soil carbon sequestration and reducing nitrogen fertilizer requirements. The paper also discusses the challenges and opportunities for mitigating GHG emissions in field cropping systems. Precision agriculture techniques, such as variable rate application of fertilizers and water, can optimize crop production while minimizing environmental impacts. Agroforestry systems, which integrate trees and crops, offer the potential for carbon sequestration and reducing N_2_O emissions. This review provides insights into the latest research on GHG emissions from field cropping systems and identifies areas for further study. It emphasizes the importance of adopting sustainable management practices to reduce GHG emissions and enhance the environmental sustainability of agricultural systems.

## 1. Introduction

Greenhouse gas emissions (GHG), primarily resulting from human activities, significantly contribute to global warming, a phenomenon evident in the Earth’s atmosphere. The concentration of greenhouse gases, such as carbon dioxide and methane, has been steadily increasing, leading to a rise in surface temperatures [1]. According to the Intergovernmental Panel on Climate Change (IPCC), the global average temperature has risen by approximately 1.1 °C (2 °F) since the late 19th century, with projections indicating an increase of an additional 2.6–4.8 °C (4.7–8.6 °F) by the end of this century [2,3]. This rise in temperature has profound implications for the planet, including more frequent and intense heat waves, droughts, floods, and wildfires [4]. Additionally, rising sea levels pose a threat to coastal communities and ecosystems. The impacts of global warming are already being felt worldwide and are expected to intensify in the future [5]. Thus, reducing greenhouse gas emissions is crucial for mitigating the effects of global warming and safeguarding the planet.

In contemporary society, human activities have significantly impacted the Earth’s climate, with the emission of greenhouse gases being a major contributor to global warming [6]. Field crop system refer to the planting and management of food and cash crops on designated plots of land, following specific temporal sequences and spatial distributions. These systems absorb carbon dioxide and release oxygen through photosynthesis while also emitting methane and nitrous oxide during growth and decomposition [7]. With the increasing severity of global warming in recent years, there has been a growing focus on studying the emission and absorption of greenhouse gases in field crop systems. Consequently, there is a rising interest in finding ways to reduce greenhouse gas emissions in these systems to help mitigate the effects of global warming.

Given the critical role that field crop systems play in both contributing to and mitigating greenhouse gas emissions, it is essential to examine how different cropping systems can influence these emissions and overall environmental impact. Cropping systems refer to the methods employed in the cultivation and management of crops. The impact of different cropping systems on greenhouse gas emissions varies based on several factors, including the types of crops cultivated, the application of fertilizers and pesticides, and the management practices related to soil and water resources. Conventional farming uses synthetic fertilizers, pesticides, and monocultures to maximize yields but can degrade soil and increase pest vulnerability [8]. Organic farming avoids synthetics, relying on natural methods like crop rotation, intercropping, and cover crops to enhance soil fertility, manage pests, and reduce erosion [9]. Monoculture involves repeated single-crop cultivation, while mixed culture and intercropping diversify plantings, improving soil health [10]. Cover crops protect soil from erosion and boost fertility [11]. Irrigated farming uses irrigation systems for water, requiring more resources, whereas dryland farming depends on rainfall [12].

Greenhouse gas emissions in field crop systems primarily arise from microbial activities in the soil and the metabolic processes of the crops [13]. Soil microorganisms decompose organic matter, generating methane and nitrous oxide, while carbon dioxide is released through redox reactions [14]. Furthermore, greenhouse gases are produced during both crop growth and decomposition [15]. Fertilization significantly contributes to increased emissions, as the excessive use of chemical fertilizers can lead to nutrient loss, ecosystem damage, and elevated residual nitrogen levels that promote nitrous oxide emissions [16]. Therefore, addressing greenhouse gas emissions in field crop systems through alterations in crop planting structures, modifications in fertilization techniques, and optimization of field management practices is a pressing concern in agriculture. The sustainable development of field crop systems can only be achieved through systematic scientific research and ongoing technological innovation, which will provide more effective solutions to combat global warming.

The study of greenhouse gases in field crop systems is crucial for evaluating the impact of climate change on agricultural production and for identifying strategies to mitigate the adverse effects of agricultural activities on greenhouse gas emissions. In light of the escalating threat of global warming and the substantial contribution of agriculture to these emissions, sustainable agricultural development has emerged as a critical concern.

Studying greenhouse gas emissions in field crop systems is essential for understanding the impact of various crops on greenhouse gas levels during their growth. The quantity of greenhouse gases emitted varies among different crop types throughout the planting and management phases [17]. By analyzing the emission characteristics of these crops, targeted measures can be implemented to effectively reduce greenhouse gas emissions. Appropriate management strategies for different crops in actual production scenarios can significantly mitigate greenhouse gas emissions [18]. Furthermore, research on greenhouse gas emissions within field crop systems can provide scientific evidence to inform the formulation of agricultural emission reduction policies. Understanding the emission profiles of various crops enables governments and relevant agencies to develop targeted policies that promote agricultural emission reduction efforts. Through scientifically sound policy-making, farmers can be guided to adopt effective emission reduction measures, thereby enhancing the sustainability of agricultural production and minimizing negative environmental impacts. Investigating greenhouse gas emissions in field crop systems ultimately contributes to environmental protection and the achievement of sustainable agricultural practices.

## 2. The Theoretical Basis of Greenhouse Gases

### 2.1. Types and Characteristics of Greenhouse Gases

In field crop systems, greenhouse gases are defined as gases that contribute to the greenhouse effect in the Earth’s atmosphere, primarily including carbon dioxide, methane, nitrous oxide, and chlorofluorocarbons (CFCs). The accumulation of these gases leads to an increase in the Earth’s surface temperature, thereby exacerbating global warming. Among them, carbon dioxide is the most significant greenhouse gas, originating from the combustion of fossil fuels and deforestation [19]. According to the IPCC, methane primarily originates from livestock and paddy field emissions and is 28 times more potent than carbon dioxide in terms of its global warming potential [20]. Nitrous oxide primarily originates from fertilizer and industrial waste gas emissions, possessing a stronger greenhouse effect than carbon dioxide [21]. CFCs, being synthetic chemicals, are currently recognized as one of the most potent greenhouse gases.

In addition to the major greenhouse gases, other gases such as water vapor, ozone, and sulfur dioxide also contribute to the greenhouse effect. Water vapor, the most abundant greenhouse gas, is naturally regulated and is not easily influenced by human activities. While ozone is typically harmful at the Earth’s surface, it functions as a greenhouse gas in the atmosphere. Sulfur dioxide, although having a weaker greenhouse effect, can oxidize to form sulfuric acid gas, which exhibits a potent greenhouse effect [22]. The sources of these greenhouse gases vary, resulting in differing impacts on field crop systems. Numerous types of greenhouse gases are present in field crop systems, with emissions influenced by both human activities and natural factors. In double cropping rice systems, an optimal fertilization strategy can account for low climate impact, high nitrogen agronomic use efficiency, and no adverse influence on yield [23]. In Mediterranean agricultural systems, management strategies, including adjustments to fertilization practices, the use of organic fertilizers, the application of urease and nitrification inhibitors, and the optimization of irrigation methods can effectively mitigate greenhouse gas emissions [24]. Furthermore, structural changes such as reducing food waste and modifying dietary patterns can contribute to the overall reduction of greenhouse gas emissions. Consequently, effective management strategies aimed at reducing the adverse effects of these gases on field crop systems must comprehensively consider various factors and implement emission control and mitigation measures.

Greenhouse gases, such as carbon dioxide, methane, nitrous oxides, and CFCs, have a significant impact on Earth’s atmosphere due to their effect [25]. These gases possess distinct physicochemical properties that influence the planet’s climate. Carbon dioxide stands out as a major greenhouse gas due to its ability to absorb and re-radiate infrared radiation from the Earth’s surface [25]. Methane, on the other hand, is characterized by being lighter than air, flammable, and soluble in water. Nitrous oxides exhibit a strong greenhouse effect and can influence redox reactions in the atmosphere. Lastly, CFCs are synthetic gases known for their resistance to absorption by other atmospheric gases, resulting in a prolonged presence in the atmosphere. Overall, the diverse physicochemical properties of greenhouse gases contribute significantly to climate change.

### 2.2. Greenhouse Effect and Its Environmental Impact

The concentration of greenhouse gases in the atmosphere is increasing, contributing to global warming and rising sea levels. This intensification of the greenhouse effect significantly impacts cropping systems, influencing growth cycles, yields, and the quality of crops [26]. Therefore, it is essential to reduce greenhouse gas emissions and mitigate global warming to support agricultural production. Strategies to decrease emissions include promoting green energy, encouraging low-carbon lifestyles, and enhancing the monitoring and management of greenhouse gases.

The multifaceted impacts of greenhouse gas emissions on the environment are intricate and severe. Firstly, the increase in greenhouse gas emissions is leading to changes in the Earth’s climate, resulting in rising temperatures and more frequent extreme weather events [27]. These changes have a detrimental effect on the growing conditions of crops, impacting both their yield and quality. Secondly, greenhouse gas emissions also have an impact on water resources, causing either a shortage of supply or pollution of water, which in turn affects crop irrigation and growth [28]. Additionally, these emissions alter the pH and nutrient content of the soil, influencing crop absorption and utilization, ultimately leading to reduced yield or quality [29]. In conclusion, the impact of greenhouse gas emissions on cropping systems is extensive, affecting the growing environment, water resources, and soil quality negatively. Therefore, effectively reducing greenhouse gas emissions is crucial for ensuring the healthy development of cropping systems.

### 2.3. Field Crop Systems and Climate Change

The response of crop systems to climate change is a complex and multi-layered process. Climate change directly affects the crop growth environment by influencing factors like temperature, precipitation, and sunlight. Global warming can result in reduced yields for some crops due to alterations in their optimal growth conditions [30]. Furthermore, extreme weather events caused by climate change, such as droughts and floods, have a severe impact on crop growth [31]. In addition, climate change can contribute to outbreaks of crop pests and diseases, further affecting crop yield and quality.

The response of crop systems to climate change encompasses not only adaptation to the growth environment but also the intrinsic growth characteristics of the crops themselves. Certain crop varieties may exhibit a capacity to adapt to the environmental changes induced by climate change. For instance, crops with robust cold resistance are likely to perform better under cooler conditions [32]. Furthermore, the cultivation and management of crops are significantly influenced by climate change. Greenhouse gas emissions affect the atmospheric environment, which subsequently impacts crop growth and yield. The response of crop systems to climate change is a multifaceted process involving numerous factors and layers, necessitating further in-depth research into the adaptability of crops to climate change, as well as the development of strategies to enhance their disaster resistance and overall adaptability. By implementing scientifically informed planting management practices and reducing greenhouse gas emissions, the productivity of crop systems can be better safeguarded and improved, ultimately contributing to sustainable agricultural development.

Greenhouse gas emissions in field crop systems are a complex and significant area of research. Crop growth, soil respiration, fertilization, and irrigation in farmland ecosystems all affect greenhouse gas emissions. During rice growth, methane is produced by soil microorganisms and released into the atmosphere through gas exchange [33]. Additionally, the decomposition processes in paddy fields release substantial amounts of methane. Besides methane, carbon dioxide is another critical greenhouse gas in field crop systems. Crop respiration and the decomposition of organic matter release carbon dioxide. The extensive use of fertilizers in agriculture also leads to the emission of nitrogen oxides from the soil, exacerbating greenhouse gas emissions [34]. Soil nitrogen oxides and ferric oxide significantly affect the conversion of nitrogen oxides, making the understanding of soil microbial ecology essential for reducing nitrogen oxide emissions [35].

Greenhouse gas emissions in field crop systems are influenced by both natural and human factors. Improper management practices and excessive fertilization can lead to increased emissions. To mitigate these emissions, it is essential to optimize management practices, reduce fertilizer usage, enhance soil quality, and improve crop production efficiency. This multifaceted approach seeks to achieve a balance between ecological and economic benefits. Overall, addressing greenhouse gas emissions in field crop systems necessitates a comprehensive understanding of both natural and human factors to develop scientifically sound management strategies that align with sustainable development goals. By conducting in-depth research on the mechanisms and factors influencing greenhouse gas emissions in field ecosystems, valuable scientific evidence can be generated to support emission reduction efforts, thereby advancing environmental protection and fostering sustainable agricultural development.

## 3. Greenhouse Gas Research Methods

### 3.1. Greenhouse Gas Monitoring Technology

With the escalating severity of global climate change, there has been a growing emphasis on greenhouse gas monitoring technology. The primary methods currently employed for monitoring greenhouse gases include traditional gas sampling and analysis, spectroscopic detection, and sensor technology.

Gas sampling and analysis technology is a widely used method for monitoring greenhouse gases. This technique involves collecting atmospheric samples and analyzing them in the laboratory with instruments such as gas chromatographs and mass spectrometers to determine the concentrations of various gas components [36]. Despite its effectiveness, this method has limitations, including uneven sampling and complex operational procedures, which impede continuous and real-time monitoring.

Spectroscopic detection technology is a method employed to monitor greenhouse gases by analyzing the absorption characteristics of gas molecules in relation to light. Instruments such as infrared spectrometers and UV-visible spectrometers are utilized to directly detect the absorption spectra of various gas molecules, thereby providing insights into their components and concentrations (Table 1). This method is highly accurate and is widely regarded as a preferred choice for monitoring greenhouse gas emissions in field crop systems.

Recent advancements in sensor technology have significantly enhanced greenhouse gas monitoring capabilities. By deploying sensors in diverse locations, real-time monitoring of atmospheric greenhouse gases is now achievable. The compact size and rapid response of these sensors render them particularly suitable for large-scale gas monitoring applications [37]. Nonetheless, there remains a pressing need to further enhance the accuracy and stability of sensor technology to fulfill the demands of high-precision and long-term monitoring.

Traditional gas sampling and analysis technology, spectroscopic detection technology, and sensor technology each offer unique strengths and weaknesses, allowing for the selection of the most suitable technology based on specific monitoring requirements. As technology continues to advance and improve, greenhouse gas monitoring technology is expected to offer increasingly precise and efficient support for the management and protection of field crop systems.

### 3.2. Data Analysis Method

Data analysis is a critical component of research methodologies. This review involved the collection, cleaning, and organization of greenhouse gas emission data from field crop systems to ensure both reliability and accuracy [38]. Descriptive statistical methods, including mean, standard deviation, maximum, and minimum values, were employed to elucidate the distribution characteristics of the data [39]. In addition to descriptive statistical analysis, regression analysis was performed to explore the relationship between greenhouse gas emissions in field crop systems and various influencing factors [40]. Mathematical models were developed to quantify the impact of these factors on greenhouse gas emissions, thereby revealing underlying patterns [41]. Furthermore, time series analysis was utilized to examine trends in greenhouse gas emissions over time, analyzing periodicity and trends to provide insights for future prediction and control.

Spatial analysis methods were employed to investigate greenhouse gas emissions in field crop systems, utilizing Geographic Information System (GIS) technology to enable spatial overlay analysis [42]. By integrating geographic location information with emission data, this approach revealed the spatial distribution patterns of greenhouse gas emissions across diverse regions, facilitating the identification of regional disparities [43]. Furthermore, through the integration of various data analysis techniques, a more comprehensive understanding of greenhouse gas emissions in field crop systems can be achieved, providing a solid scientific foundation and valuable insights for future research and management endeavors.

Life cycle assessment (LCA) is a widely employed method for quantifying the GHG emissions associated with a product or service throughout its entire life cycle (Figure S1). LCA considers emissions at all stages, including raw material extraction and processing, manufacturing, distribution and transport, usage, and final waste treatment and recycling [44]. This comprehensive assessment method provides a thorough understanding of the environmental impact of a product or service over its life cycle, aids in identifying major emission sources and potential reduction opportunities, and thus assists companies and decision-makers in developing sustainable development strategies [45]. By leveraging LCA analysis, relevant stakeholders can gain insights into the environmental performance of products or services, thereby fostering more environmentally friendly design and production practices, ultimately contributing to a reduction in climate change impacts [46].

### 3.3. Model Simulation and Prediction

In field crop systems, model simulation and prediction can improve our understanding and prediction of greenhouse gas emissions. By developing mathematical models, we can simulate greenhouse gas emissions throughout crop growth and make projections for future emissions. To establish reliable models, a significant amount of experimental data, such as meteorological data, soil properties, and crop varieties, needs to be collected. This data allows us to assess the impact of various factors on greenhouse gas emissions and combine them using mathematical formulas to create a comprehensive simulation system [47]. Additionally, model validation and adjustment are crucial steps. Historical data can be utilized for model validation to compare simulation results with actual data [48]. If inconsistencies are found, adjustments to the model are necessary to enhance its accuracy and reliability.

Models can also be utilized to predict future greenhouse gas emissions by incorporating potential future meteorological data and crop planting schemes. This allows us to anticipate future emissions and develop appropriate policies and strategies to mitigate greenhouse gas emissions. Model simulation and prediction serve as valuable tools in enhancing our understanding and management of greenhouse gas emissions in field crop systems. Through ongoing enhancements and refinements to these models, we can effectively safeguard the environment and progress toward achieving sustainable development objectives.

## 4. Greenhouse Gas Emission Dynamics

### 4.1. Seasonal Variation of Emission Flux

In spring, rising temperatures and longer daylight hours lead to increased microbial activity in the soil, resulting in higher greenhouse gas emissions [49]. Among these emissions, nitrogen oxides play a significant role. Nitrogen oxides primarily stem from soil nitrogen cycle processes such as ammonia oxidation and nitrification. The emission of nitrogen oxides during spring is largely influenced by soil temperature and moisture levels. Higher temperatures and moisture content lead to greater nitrogen oxide emissions [50]. Methane, another key component of spring and summer greenhouse gas emissions, is mainly produced by flooded crops like rice paddies [51]. As spring is the main season for rice planting, methane emissions from rice paddies also increase [52]. Methane emissions are impacted by moisture and oxygen levels, with anaerobic conditions in rice paddies fostering microbial production of methane, which is further stimulated by heavy rainfall in spring [53]. Additionally, carbon monoxide, originating from combustion processes like biomass burning and vehicle exhaust, is another significant greenhouse gas emitted during spring [54]. As temperatures increase, farmers burn crop residues, and vehicle usage rises, resulting in a notable spike in carbon monoxide emissions [55]. Overall, spring is a period marked by high greenhouse gas emissions in field crop systems, primarily driven by nitrogen oxides, methane, and carbon monoxide. Future research should focus on understanding the dynamic changes in spring greenhouse gas emissions to develop effective mitigation strategies and minimize the impact of climate change.

**Figure 1:**
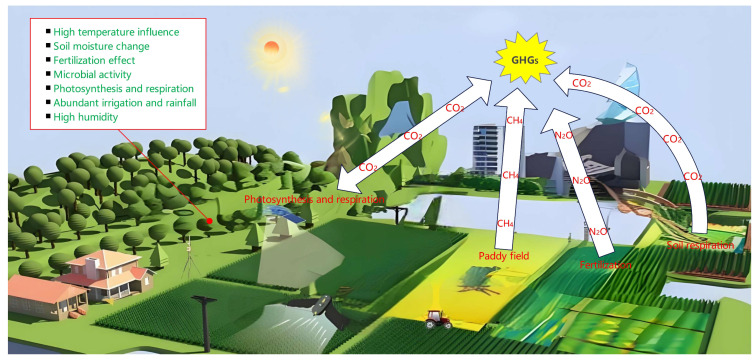
Greenhouse gas flux in summer field crop system.

Summer plays a pivotal role in greenhouse gas emissions within field crop systems, driven by elevated temperatures, intense sunlight, and substantial rainfall impacting crop growth and emissions (Figure 1). The primary greenhouse gases involved in the atmosphere include carbon dioxide, methane, and nitrous oxide. During summer, carbon dioxide emissions may peak due to heightened temperatures stimulating plant photosynthesis, which absorbs and releases carbon dioxide [56]. The rise in plant respiration during periods of elevated temperatures significantly increases carbon dioxide emissions. Similarly, the summer season is critical for methane emissions, as the combination of abundant irrigation and rainfall in environments such as rice paddies creates anaerobic conditions that favor methane production. The synergy of high temperatures and moisture levels during summer further stimulates the growth of methane-producing microbial communities, thereby amplifying emissions [57]. Additionally, nitrous oxide emissions exhibit marked seasonal variation, peaking in summer. These emissions arise from microbial processes involving nitrogen in the soil, where warm temperatures, high humidity, and rapid crop growth collectively enhance microbial activity and nitrogen metabolism, resulting in increased nitrous oxide emissions [58]. Understanding the seasonal dynamics of greenhouse gas emissions during the summer months is essential for developing effective strategies to mitigate these emissions, reduce the carbon footprint of crop production systems, and promote sustainable agricultural practices.

In field crop systems, greenhouse gas emissions exhibit noticeable seasonal variations, with autumn playing a significant role as a transitional season that impacts emissions. Autumn signifies the end of the crop growth cycle, with decreasing temperatures and falling leaves directly affecting greenhouse gas emissions. During autumn, greenhouse gas emissions in field crop systems are primarily influenced by biochemical processes [59]. As the crop growth cycle approaches its end, plant respiration weakens, leading to a decrease in carbon dioxide emissions and a corresponding decline in greenhouse gas flux [60]. Moreover, reduced microbial activity in the soil due to lower temperatures further contributes to lowering greenhouse gas emissions. Additionally, the decomposition of plant residues during autumn affects greenhouse gas emissions in field crop systems. The gradual fall of crop leaves and withering root systems release methane and other greenhouse gases during decomposition, with increased atmospheric humidity accelerating this process [61]. Overall, autumn is characterized by a gradual decrease in greenhouse gas emissions in field crop systems, driven by biochemical processes and plant residue decomposition. However, the impact of climate warming and human activities on autumn greenhouse gas emissions may necessitate further research and monitoring to comprehend emission trends better.

### 4.2. Emission Differences among Different Crops

Cereal crops play a significant role in agricultural production and have a notable impact on greenhouse gas emissions. In field crop systems, greenhouse gas emissions from cereal crops primarily stem from soil organic matter decomposition and the crops’ growth processes [62]. Rice and wheat show differences in greenhouse gas emissions [63]. Rice, typically grown in humid environments, requires substantial irrigation and fertilization, resulting in higher methane production in the soil. Moreover, rice paddies serve as a notable source of methane emissions. Therefore, rice stands out for its methane emissions within the spectrum of greenhouse gas emissions. In contrast, wheat, cultivated in arid environments, has lower water requirements compared to rice. Consequently, it can be inferred that wheat emits less methane than rice. However, wheat may produce higher nitrous oxide emissions during its growth cycle due to significant nitrous oxide release from fertilization [64]. Considering the greenhouse gas emission characteristics of rice and wheat, it is evident that crop selection plays a pivotal role in mitigating greenhouse gas emissions. By making informed choices regarding crops and implementing scientific fertilization and water-efficient irrigation practices, greenhouse gas emissions from cereal crops can be effectively reduced, thereby contributing to both agricultural productivity and environmental preservation.

In field crop systems, there are noticeable variations in greenhouse gas emissions across different economic crops. These differences arise from carbon emissions during crop growth and the efficiency of nutrient utilization, such as nitrogen and phosphorus, in the soil [65]. As a water-intensive crop, rice requires a lot of water resources, and the decomposition of rice straw in the paddy field releases a lot of methane, resulting in higher greenhouse gas emissions than wheat [66]. Economic crops also exhibit distinct differences in fertilization and pesticide usage [67]. Maize requires large amounts of nitrogen fertilizer during its growth, leading to emissions of nitrogen oxides and increasing greenhouse gas levels in the atmosphere [68]. On the other hand, soybeans, with their nitrogen-fixing ability, do not need external nitrogen fertilizer, thus reducing greenhouse gas emissions [69]. Moreover, the duration of the growth cycle of economic crops influences their greenhouse gas emissions, with crops having longer growth cycles generally emitting higher levels [17]. Maize has a longer growth cycle than soybeans and requires more nutrients and pesticides, leading to increased greenhouse gas emissions [70]. Overall, there are substantial disparities in greenhouse gas emissions among economic crops, primarily due to their growth characteristics and agricultural practices like fertilization and pesticide use. Future cultivation of economic crops should prioritize reducing greenhouse gas emissions through effective measures, thereby promoting sustainable agricultural development.

Green manure crops reduce greenhouse gas emissions by improving soil structure and nutrient content. These crops exhibit high photosynthetic efficiency, absorbing significant amounts of carbon dioxide for photosynthesis and releasing oxygen, thereby reducing atmospheric greenhouse gas levels [71]. Soybeans and alfalfa grow quickly and efficiently, absorb nutrients such as nitrogen and phosphorus from the soil, and store them in plant tissues [72]. These nutrients are then converted into organic compounds like proteins, reducing soil nutrient depletion and decreasing the reliance on chemical fertilizers, consequently lowering nitrous oxide and nitrate emissions [73]. They release beneficial microorganisms that facilitate organic matter decomposition and nitrification processes in the soil, thereby reducing methane and nitrous oxide emissions [74]. Additionally, the roots contribute to soil stability and water retention, minimizing oxidative conditions and nitrate formation [75]. Planting green manure crops in field crop systems is an eco-friendly and efficient agricultural practice. By maximizing the utilization of green manure crops, it is possible to effectively reduce greenhouse gas emissions, enhance soil fertility and structure, and achieve sustainable agricultural production. Future agricultural strategies should prioritize the widespread adoption of green manure crops in field crop systems to promote more environmentally friendly and sustainable production methods.

### 4.3. Impact of Farmland Management Practices

The selection of irrigation methods is crucial in farmland management practices and greenhouse gas emissions. It not only affects water use efficiency in farmland but also influences the transformation and loss of nitrogen, carbon, and other elements in the soil [76]. Irrigation can significantly reduce GHG emissions compared to dryland farming, with a 20–30% reduction in CO_2_ equivalents observed in irrigated systems [77]. Flood irrigation can lead to the leaching of nitrogen, carbon, and other elements in the soil, resulting in increased greenhouse gas emissions [78]. Drip irrigation systems can help reduce water wastage, but improper fertilization and irrigation management may still cause nitrogen and carbon loss. Modern irrigation methods such as micro-irrigation systems and precision irrigation techniques have shown excellent performance in reducing nitrogen and carbon loss in the soil. Micro-irrigation systems deliver water directly to plant roots through irrigation pipes, minimizing water loss and nitrogen and carbon leaching in the soil [79]. Precision irrigation techniques use meteorological data, soil moisture levels, and crop water requirements to implement precise irrigation tailored to the specific water needs of plants [80]. This not only improves crop yield but also reduces nitrogen and carbon loss, ultimately lowering greenhouse gas emissions.

Subsurface drip irrigation and infiltration irrigation are two common methods used in farmland management. Subsurface drip irrigation involves a pipe system that delivers water underground, reducing water, nitrogen, and carbon loss in the soil. In contrast, seepage irrigation incorporates pores on irrigation pipes to allow water to slowly seep into the soil, improving soil water use efficiency and reducing nitrogen and carbon loss [81]. The choice of irrigation methods is crucial in reducing greenhouse gas emissions from farmland. When selecting irrigation methods for farmland management, it is important to consider water use efficiency and the potential loss of nitrogen and carbon in the soil to prevent increased greenhouse gas emissions from excessive fertilization and improper irrigation practices. By strategically choosing irrigation methods, greenhouse gas emissions from farmland can be effectively minimized, supporting sustainable agricultural development.

Fertilization strategies are crucial in field crop systems, with different methods having varying impacts on greenhouse gas emissions. Traditional chemical fertilization often results in waste and poor absorption, leading to excessive nitrogen accumulation in the soil and subsequent greenhouse gas emissions [82]. Conversely, the use of organic materials can decrease the reliance on chemical fertilizers, addressing the issue of nitrogen accumulation and reducing emissions [83]. The type and quantity of fertilizer used are significant factors influencing greenhouse gas emissions. Studies indicate that excess nitrogen fertilizer can lead to increased emissions of nitrous oxide and methane due to residual nitrogen [84]. Therefore, a scientific approach to fertilization based on crop requirements is essential to prevent the overuse of nitrogen fertilizers and mitigate the negative effects on greenhouse gas emissions.

Fertilization strategies should consider soil health and fertility. Maintaining soil fertility can improve crop absorption efficiency and reduce fertilizer loss. Practices like incorporating organic matter and using mulching can enhance soil aeration and water retention, ultimately decreasing nitrogen volatilization, leaching, and nitrogen oxide emissions [85]. By carefully managing the type and amount of fertilizers and preserving soil health, greenhouse gas emissions can be minimized, reducing the environmental impact of agricultural land and promoting sustainability. Therefore, the development and implementation of fertilization strategies in field crop systems are crucial and require further research and discussion.

Changes in tillage methods have a significant impact on greenhouse gas emissions in field crop systems. Different tillage methods can either enhance or reduce soil carbon storage. Traditional farming methods can damage soil structure and lead to carbon release [86]. On the other hand, no-till or conservation tillage helps preserve soil carbon stocks and reduce carbon emissions [87]. Moreover, variations in tillage methods also impact microbial activity in the soil. Microorganisms influence greenhouse gas emissions in the decomposition of organic matter and soil carbon cycle [88]. By adjusting tillage methods, the composition and abundance of soil microorganisms can be modified, subsequently affecting greenhouse gas emissions.

Changes in tillage methods can have a significant impact on nitrogen fertilizer use efficiency, which is crucial for effective farmland management. Excessive use of nitrogen fertilizers can lead to increased nitrogen emissions and, subsequently, higher levels of greenhouse gas emissions [89]. The combination of tillage and stubble return can improve nitrogen use efficiency, ultimately reducing nitrogen emissions and reducing greenhouse gas emissions [16]. The selection of appropriate tillage methods plays a key role in influencing greenhouse gas emissions within field crop systems. By choosing the right tillage methods, it is possible to effectively decrease greenhouse gas emissions and promote sustainable agricultural development.

### 4.4. Meteorological Effect

These conditions impact greenhouse gas concentrations in the atmosphere, as higher temperatures can promote emissions [90]. Furthermore, meteorological factors affect plant growth, metabolic processes, and the activity of soil microbial communities, ultimately influencing the release of greenhouse gases [91]. Understanding the role of meteorological conditions in field crop systems is essential for managing greenhouse gas emissions.

Temperature and humidity are key factors in influencing greenhouse gas emissions within meteorological conditions. Elevated temperatures can accelerate plant photosynthesis and respiration, resulting in increased carbon dioxide emissions [92]. Additionally, high temperatures can stimulate root respiration, leading to the release of methane and nitrous oxide from the soil [93]. Changes in humidity levels also impact greenhouse gas emissions, as high humidity can enhance microbial decomposition in the soil, thereby increasing the release of methane and nitrous oxide [94]. Therefore, a comprehensive understanding of how meteorological conditions affect greenhouse gas emissions is essential for the development of effective control strategies.

Meteorological conditions are closely linked to factors such as wind speed and sunlight hours. Higher wind speeds help disperse and weaken greenhouse gases, lowering their concentration in the atmosphere. Sufficient sunlight hours can boost plant photosynthesis, leading to a decrease in atmospheric carbon dioxide levels [95]. Managing meteorological conditions is vital for regulating greenhouse gas emissions in field crop systems. It is crucial to monitor and study meteorological conditions and to implement suitable management strategies in order to reduce greenhouse gas emissions and promote sustainable agricultural development.

### 4.5. Effects of Soil Properties

The content of soil organic matter has a notable impact on emissions, with higher levels typically leading to increased methane release due to the decomposition process [88]. Additionally, organic matter content affects microbial activity, influencing overall greenhouse gas emissions [96]. Soil pH is another key factor, with acidic soils generally releasing more carbon dioxide as organic matter decomposition under acidic conditions produces more carbon dioxide [97]. Soil pH also impacts the nitrogen cycle, further influencing nitrous oxide and methane emissions.

Soil texture is also significant, as soils with higher gravel content tend to emit less nitrous oxide due to gravel slowing down release rates. Furthermore, heavier textured soils limit oxygen infiltration, affecting microbial activity and, subsequently, greenhouse gas production [98]. Overall, soil properties greatly influence greenhouse gas emissions in field crop systems. Therefore, managing factors like soil organic matter content, pH, and texture are essential to reduce emissions and achieve environmental protection and sustainable agricultural development goals.

### 4.6. Association of Microbial Activity

**Figure 2:**
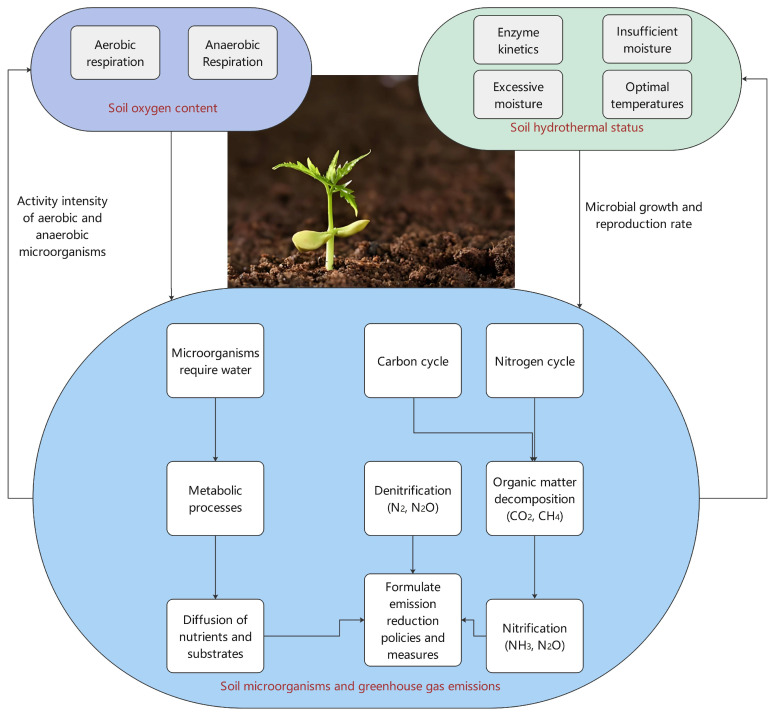
Effects of environmental factors on decomposition of organic matter and microbial activity: combined effects of soil moisture, temperature, and oxygen content. Climate change impact: Rising global temperatures can accelerate decomposition in some regions, leading to increased carbon dioxide emissions. However, changes in precipitation patterns and soil moisture can create conditions that either enhance or inhibit microbial activity. Ecosystem productivity: Efficient decomposition releases nutrients essential for plant growth, affecting primary productivity and ecosystem health. Carbon cycle: Understanding these dynamics is crucial for predicting how changes in environmental conditions will impact the carbon cycle and feedback mechanisms in the context of global climate change.

Microbial activity in field crop systems significantly influences greenhouse gas emissions as soil microorganisms decompose and synthesize organic matter, releasing CO_2_, methane, and nitrous oxide (Figure 2). The level of microbial activity directly impacts the amount of greenhouse gas emissions from the soil [99]. Bacteria and fungi in the soil significantly influence microbial activity, with bacteria primarily releasing CO_2_ through organic matter decomposition, while fungi decompose more recalcitrant organic matter, producing methane [100]. The number and diversity of microorganisms in the soil also directly affect microbial activity strength, thereby influencing greenhouse gas emissions.

Environmental factors such as soil moisture, temperature, and oxygen content also impact microbial activity by affecting the growth and reproduction rate of microorganisms, thus influencing organic matter decomposition. Studies have shown that microbial activity varies with temperature conditions, being lower in winter and increasing in summer. Agricultural activities directly impact soil microbial activity, with the amount and type of fertilizers applied influencing the number and diversity of soil microorganisms, thus affecting greenhouse gas emissions. Effective management of soil microbial activity in field crop systems is crucial for reducing greenhouse gas emissions. Measures like optimizing fertilization schemes and improving soil aeration conditions can effectively reduce microbial greenhouse gas emissions, thereby mitigating agriculture’s negative impact on climate change.

## 5. Construction of Greenhouse Gas Emission Model

### 5.1. Model Framework and Parameterization

The fundamental principle of greenhouse gas emission models is to simulate and predict emissions by analyzing crop growth processes, soil carbon cycling mechanisms, and meteorological conditions. The model’s theoretical basis includes key processes like soil respiration, crop transpiration, and radiation absorption [101]. Soil respiration involves soil microorganisms decomposing organic matter, releasing carbon dioxide. Crop transpiration is the release of water vapor through leaf surfaces, while solar radiation absorption by crops influences greenhouse gas emissions [102]. Parameterization entails setting and adjusting various model parameters like soil organic carbon content, temperature, humidity, and light. The accuracy and appropriateness of these parameters significantly impact the model’s predictive ability [103]. Therefore, thorough research and analysis of these parameters are essential in constructing greenhouse gas emission models. The model framework design should also account for variations in different climate and soil conditions, as crop systems in various regions are influenced by diverse meteorological conditions and soil characteristics. Hence, adjustments and optimizations based on regional specifics are crucial.

**Figure 3:**
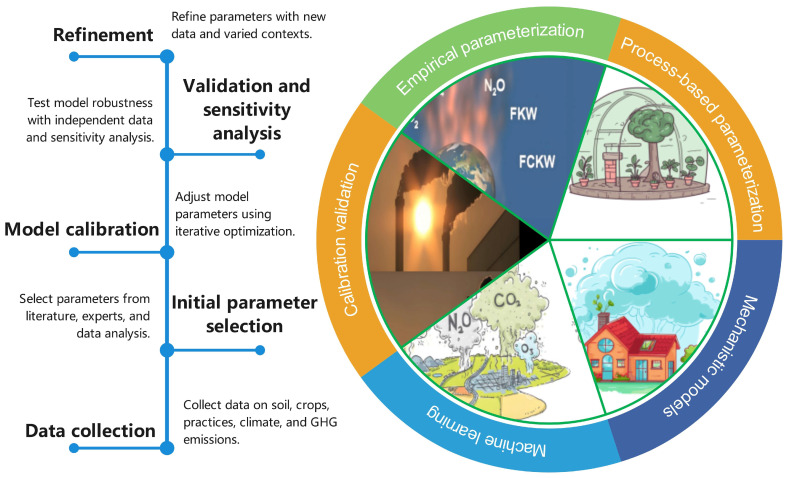
Application of multi-method parameterization and calibration model to GHG emission prediction. Empirical parameterization: Uses observed data to establish variable relationships, such as soil moisture, temperature, and crop yield, to predict GHG emissions. Process-based parameterization: Relies on biological, chemical, and physical processes driving GHG emissions, including soil nitrogen dynamics, plant growth, and microbial activity. Mechanistic models: Incorporate detailed mechanisms of GHG production and consumption, with parameters for nitrification, denitrification, and carbon mineralization. Machine learning: Utilize advanced techniques to identify patterns and parameterize models from large datasets, handling non-linear relationships and high-dimensional data. Calibration and validation: Involves tuning models with a subset of data and validating against independent datasets to ensure accurate real-world representation.

The parametric method is helpful in constructing a greenhouse gas emission model of a field crop system (Figure 3). This process entails translating abundant observational data and experimental findings into model-specific parameters to enhance the accuracy and dependability of simulating greenhouse gas emissions in field crop systems. The steps involved in parameterization include identifying key parameters throughout the growth stages of chosen field crops, such as soil temperature and moisture, vegetation cover, and climatic conditions. Subsequently, these parameters are quantified through field sampling and analysis and then transformed into a format compatible with the model.

During the parameterization process, it is crucial to appropriately allocate weights to different factors to simulate greenhouse gas emissions accurately under varying conditions. Parameterization methods should also account for the interplay between factors in field crop systems, such as energy and material exchanges among soil, vegetation, and the atmosphere [104]. Furthermore, the steps involved in parameterization methods entail a thorough analysis of existing research findings and models to identify essential parameters and determine which ones can be disregarded. Through the verification and adjustment of existing models and data, parameterization methods can be continually refined and optimized to improve the model’s practicality and predictive accuracy.

A crucial step in developing greenhouse gas emission models is the identification of sensitive parameters. These parameters have a significant impact on the model’s output, with their variations leading to substantial changes in the results. Recognizing sensitive parameters enables more precise predictions of greenhouse gas emissions [105]. Common approaches for pinpointing sensitive parameters include global sensitivity analysis and local sensitivity analysis. Global sensitivity analysis involves perturbing all parameters in the model through a series of experiments or numerical simulations to observe changes in the output, thereby determining the influence of each parameter. On the other hand, local sensitivity analysis focuses on specific parameters, examining changes in the output by adjusting the values of those parameters [106]. Historical data or experimental results can be utilized to validate sensitive parameters in the model, enhancing its accuracy and reliability. By employing sound sensitivity analysis techniques and considering the interactions between parameters, sensitive parameters can be identified more accurately, offering robust support for precise prediction of greenhouse gas emissions in field crop systems.

### 5.2. Model Validation and Calibration

The accuracy and reliability of a model are heavily dependent on the validation data collected. It is crucial to gather meticulous and comprehensive data to ensure the model’s performance. The selection of validation data types and metrics should be based on the model’s characteristics and requirements. These metrics may encompass factors like surface temperature, crop growth conditions, and meteorological data to provide a comprehensive reflection of real-world scenarios [107]. Data authenticity and reliability must be prioritized throughout the data collection process, with a focus on minimizing human interference and operational errors to maintain data integrity and accuracy. The use of modern sensors and monitoring equipment can aid in real-time data monitoring, reducing the influence of human factors on the collected data [108].

When collecting validation data, it is important to consider the spatiotemporal distribution of the data. Data from different regions and seasons may vary, so it is necessary to comprehensively analyze data from all aspects to achieve more accurate validation results [109]. Assessing the applicability and accuracy of the model can be enhanced by examining the spatiotemporal distribution of the data. It is crucial to also take into account the updating and dynamism of the data during validation data collection. As time progresses, various factors may change, requiring prompt updates to the validation data to maintain the model’s accuracy and effectiveness. Continuously improving and updating validation data by combining real-time monitoring data with historical data can enhance the predictive capabilities of the model [110].

The efficiency and applicability of a model are crucial factors in evaluating its quality. Efficiency pertains to the model’s speed and resource usage, impacting work productivity. Applicability refers to how well the model can be used in diverse scenarios, accurately reflecting greenhouse gas emissions in different conditions. A highly efficient model often has high applicability and is able to swiftly handle numerous calculations and analyses. To ensure accuracy, the model should be compared to actual observation data, with continuous validation and calibration being essential for improvement. By developing an efficient and widely applicable model, agricultural producers and policymakers can gain valuable insights into managing greenhouse gas emissions in field crop systems.

Calibration is a critical step in developing greenhouse gas emission models, as the choice of calibration methods directly impacts the model’s accuracy and reliability. In field crop systems, greenhouse gas emissions are influenced by factors such as climatic conditions, soil types (sandy soils, loamy soils, clay soils, peat soils), and crop types. Accurate data collection, including meteorological, soil, and crop growth data, is essential. Calibration parameters, such as emission and absorption rates of greenhouse gases, need to be carefully selected. The calibration process often involves multiple iterations to adjust model parameters by comparing them with actual observation data, aiming to improve the model’s predictive accuracy [111]. During calibration, uncertainties should be taken into account to minimize errors and enhance the model’s precision. Simulation experiments can help validate the model’s accuracy by controlling environmental conditions and comparing the model’s predictions with experimental data. Continuous adjustment of model parameters can lead to closer alignment between the model’s predictions and actual conditions.

### 5.3. Model Application and Scenario Analysis

Models are valuable tools for evaluating the impact of different crop types and planting patterns on greenhouse gas emissions. They can quantitatively assess how various planting methods and management practices influence emissions, as well as simulate the effects of climate change on crop growth and subsequent greenhouse gas emissions [112]. By optimizing crop planting structures and management practices through simulation and analysis, models can help identify the most effective strategies for minimizing emissions. This is essential for achieving emission reduction targets and promoting sustainable development. Expanding the application of models can provide valuable guidance for managing field crop systems, meeting emission reduction goals, protecting the environment, and fostering sustainable development.

In field crop systems research, it is crucial to first define the research object, including crop types, planting area, and fertilization amounts. External factors like climate conditions, soil properties, and management practices that influence greenhouse gas emissions should also be taken into account [113]. By simulating emissions under various scenarios, differences can be compared to identify key factors and their potential impacts [113]. It is important to ensure the reliability of model parameters and results by using field survey data and the literature to set parameters and validate simulation outcomes against measured data. This systematic approach can provide reliable data on greenhouse gas emissions, serving as a scientific basis for emission reduction strategies.

By utilizing established models, the impact of various agricultural activities on greenhouse gas emissions can be assessed with greater accuracy, providing a scientific basis for the development of effective emission reduction strategies. It is essential to thoroughly interpret and analyze the results obtained from these models. During this interpretation process, a comprehensive consideration of factors such as nitrogen fertilizer application rates, soil types, and crop species is necessary to understand their collective influence on greenhouse gas emissions [114]. Through an in-depth analysis of data patterns, the underlying rules and influencing factors of greenhouse gas emissions can be elucidated more clearly. Given the current emphasis on promoting low-carbon agricultural practices, the potential applications of greenhouse gas emission models are extensive. By simulating emissions under various agricultural production scenarios, these models can offer valuable recommendations for emission reductions to governmental bodies and farmers, thereby facilitating the transition towards low-carbon and high-efficiency agricultural production methods.

### 5.4. Model Uncertainty and Suggestions for Improvement

Uncertainty in observational data is a key contributor to model uncertainty, influenced by factors like instrument precision, sampling frequency, and environmental variability [115]. When developing greenhouse gas emission models, it is crucial to process and calibrate observational data effectively to minimize errors. The structural uncertainty of these models, based on theoretical assumptions and mathematical derivations, also plays a significant role in model accuracy. Assumptions about climate change trends can lead to deviations from reality and introduce uncertainty into model outcomes. Parameter uncertainty, stemming from estimating parameters like emission factors and ecosystem parameters, is another source of model error [116]. Measurement errors and estimation methods can impact parameter accuracy, leading to uncertainty in parameter estimation [117]. Techniques such as sensitivity analysis and parameter optimization can help mitigate parameter uncertainty by adjusting and optimizing these parameters.

Researchers can explore various avenues to enhance greenhouse gas emission models. Firstly, there is a pressing need to improve monitoring and research on greenhouse gas emission factors, particularly by analyzing in greater detail the influences of environmental conditions on emissions during crop growth. Secondly, it is essential to strengthen the study of the emission characteristics of different field crops by investigating how various planting, fertilization, and irrigation methods affect emissions. The utilization of modern technologies, such as remote sensing and meteorological models, can facilitate more accurate monitoring and simulation of greenhouse gas emissions in field crop systems, thereby increasing the precision and reliability of these models [118]. Furthermore, researchers can focus on developing greenhouse gas emission prediction models that leverage machine learning and artificial intelligence technologies, utilizing big data analysis and algorithm optimization to enhance the predictive capacity for greenhouse gas emissions.

Long-term monitoring is essential for obtaining more accurate data to validate and refine models. By conducting long-term monitoring, researchers can gather comprehensive and realistic experimental data, thus enhancing the accuracy and reliability of the model. Additionally, monitoring helps in gaining a deeper understanding of the mechanisms and patterns of greenhouse gas emissions, providing a solid foundation for model development. Model updating is a crucial process that involves continuously optimizing and enhancing the model by integrating new data and information. This ensures that the model can better adapt to real-world conditions and improves its predictive and analytical capabilities. Moreover, model updating serves as a method to address model uncertainty, gradually minimizing errors and biases through ongoing iteration and enhancement.

## 6. Evaluation of Emission Reduction Measures and Strategies

### 6.1. Analysis of Emission Reduction Measures

Conservation tillage technology reduces the use of fertilizers and pesticides, decreases the frequency of soil tillage, and protects the integrity and stability of soil ecosystems. Implementing measures such as straw returning, intercropping, and the application of organic fertilizers, as well as conservation tillage, can effectively reduce nitrous oxide emissions in fields [119]. Straw returning is a common conservation tillage technique that includes an important source of carbon-rich organic material [120]. This practice increases the soil’s organic matter content, enhances its water and nutrient retention capacity, and reduces the need for chemical fertilizers [121]. Additionally, straw returning promotes the growth of soil microorganisms, contributing to the restoration and health of the soil ecosystem. Intercropping, another crucial conservation tillage technique, entails planting different types of seasonal crops in the same field. This approach improves soil structure and fertility, slows soil degradation, and reduces emissions of nitrous oxide and methane [91]. Intercropping also helps decrease pests and diseases, leading to a reduced need for pesticides and a lower environmental impact of agricultural activities [122]. Furthermore, the application of organic fertilizers is a key aspect of conservation tillage. Organic fertilizers, with their slow-release, long-lasting effects and soil improvement properties, enhance soil fertility, improve soil quality, reduce excessive nitrogen and phosphorus fertilizer use, and lower nitrous oxide and methane emissions [123]. The use of organic fertilizers also stimulates the growth and activity of soil microorganisms, aiding in nitrogen cycling and the decomposition of organic matter [124].

Fertilization management is a critical aspect of field crop systems, as it enhances crop yield and quality while simultaneously reducing greenhouse gas emissions. The initial consideration in optimizing fertilization management should be the soil nutrient status, with soil testing serving as a valuable tool for assessing nutrient content and guiding the targeted application of appropriate fertilizers. By aligning scientific fertilization practices with the specific needs of various crops and their growth stages, the risk of nutrient waste and excessive nitrous oxide emissions can be minimized. Employing green fertilization methods, such as organic fertilizers, biofertilizers, green manure, and organic-inorganic compound fertilizers, can help mitigate the environmental impact of chemical fertilizers, improve soil fertility, and further reduce greenhouse gas emissions. Additionally, the timing and frequency of fertilization are crucial in optimizing fertilization management. Adhering to appropriate timing and frequency based on crop growth requirements and seasonal characteristics can enhance fertilizer utilization efficiency and decrease the loss of unabsorbed nutrients into the environment, ultimately leading to a reduction in gas emissions.

Crop rotation and intercropping are effective emission reduction techniques that can lower greenhouse gas emissions. Crop rotation involves planting different crops in a specific sequence on the same land, improving soil texture and increasing organic matter content, thus reducing the need for fertilizers and the release of nitrogen [125]. Intercropping involves planting two or more crops simultaneously on the same land, enhancing crop utilization efficiency and slowing the rapid release of fertilizers in the soil. Both crop rotation and intercropping can increase land use efficiency, reduce fertilizer use, and lower greenhouse gas emissions in field crop systems [126]. Research has shown that crop rotation significantly increases soil organic matter content, improves soil structure, and reduces ammonia volatilization, thereby decreasing greenhouse gas emissions [127]. Intercropping can also improve the utilization efficiency of nutrients such as nitrogen and phosphorus in the soil, reduce crop dependence on fertilizers, and lower greenhouse gas production [122]. Additionally, crop rotation and intercropping can enhance ecosystem diversity, promote soil microorganism activity, and increase soil organic matter, contributing to environmental protection and reducing farmland pollution [128]. Therefore, crop rotation and intercropping not only reduce greenhouse gas emissions but also improve land productivity, achieving sustainable agricultural development.

### 6.2. Comprehensive Emission Reduction Effect Evaluation

When evaluating the overall impact of emission reduction measures, it is crucial to consider how different strategies affect greenhouse gas emissions. Simulation experiments can be employed to assess the impact of various reduction measures on greenhouse gas emissions, specifically evaluating their effectiveness in reducing emissions through mulch and irrigation water controls. These simulations can analyze the effects of strategies on methane and nitrous oxide emissions. Gas sampling sites can be established in areas where mitigation measures are implemented, allowing for the collection of atmospheric samples to measure changes in greenhouse gas levels and assess the effectiveness of these strategies. Furthermore, monitoring gas emission fluxes in field crop systems can provide valuable insights into the actual effectiveness of the reduction measures.

Cost-benefit analysis is a crucial method for evaluating emission reduction measures in the field crop system. It involves comparing reduction costs, which include expenses for equipment investment, operation, maintenance, and energy consumption, with the benefits of assessing the rationality and feasibility of the measures [129]. In this context, the use of biofertilizers as a substitute for chemical fertilizers can increase certain production and transportation costs but lead to long-term reductions in soil nitrogen emissions, thus lowering greenhouse gas emissions [130]. The benefits of emission reduction are primarily measured by the amount of greenhouse gases reduced through the implemented measures. Reducing methane and nitrous oxide emissions through practices like reasonable fertilization, crop rotation, and planting adaptive crops can effectively lessen greenhouse gas emissions and mitigate environmental impact [94].

When evaluating the environmental and social impacts of emission reduction measures in the field crop system, it is essential to consider a wide range of effects. Reducing greenhouse gas emissions can help slow global warming, reduce natural disasters, and maintain ecosystem stability (Table 2). The implementation of reduction measures in the field crop system can also lead to a decrease in air pollutant emissions, thereby improving air quality and minimizing harm to both humans and other organisms [131]. Social effects are equally significant in evaluating reduction strategies. Lowering greenhouse gas emissions can improve overall environmental quality within society, diminish the potential impact of natural disasters on social progress, foster the growth of a green economy, create job opportunities, and enhance residents’ quality of life. Furthermore, adopting reduction measures can support sustainable agricultural practices, boost agricultural productivity, increase farmers’ incomes, and contribute to sustainable rural development. The environmental and social impacts of emission reduction measures in the field crop system not only involve mitigating environmental risks associated with greenhouse gas emissions but also entail fostering the sustainable development of the socio-economy. Therefore, future agricultural production should focus on strengthening research and implementation of reduction measures to establish a green, low-carbon agricultural production model that delivers dual benefits for both the environment and society.

### 6.3. Policy Tools and Mechanisms

The trading of greenhouse gas emission rights, as an economic instrument, involves establishing a market mechanism that assigns emission caps to various entities or industries. This facilitates the buying and selling of emission rights and is seen as an effective way to reduce greenhouse gas emissions [139]. Under this mechanism, enterprises can trade when they reach their emission limits, enabling low-emission entities to sell their excess quotas to high-emission entities, thus helping to achieve overall emission reduction goals [140].

The implementation of greenhouse gas emission rights trading necessitates the government to develop relevant policies and regulations and establish a supervisory mechanism. Emission quotas can be allocated to enterprises through auctions or other means, with emissions monitored and penalties for non-compliance [141]. By creating these policies and mechanisms, enterprises are motivated to actively reduce emissions, fostering the growth of a low-carbon economy. A comprehensive market mechanism is essential for greenhouse gas emission rights trading, where the price of emission rights is determined by market supply and demand, prompting enterprises to independently decide on emission reductions. This market mechanism can effectively encourage technological innovation, enhance emission efficiency, and reduce production costs, leading to both economic and environmental advantages.

By offering rewards or tax reductions, policy incentives can effectively motivate farmers to adopt emission reduction measures. The government can establish a carbon emission rights trading market, enabling farmers to earn carbon emission rights by reducing emissions, which they can subsequently trade for rewards [142]. Additionally, direct subsidies can be provided to encourage farmers to implement emission reduction measures, such as subsidies for purchasing efficient fertilizers or clean energy equipment. Beyond economic incentives, educational campaigns and technical support can be employed to promote emission reductions. Publicity activities can be organized to educate farmers on the significance and methods of emission reduction, thereby fostering environmental awareness. Furthermore, technical support, including training courses on emission reduction technologies and equipment, can assist farmers in implementing these measures more effectively.

Agricultural carbon sequestration projects involve implementing a series of agricultural activities to increase soil organic carbon storage, thereby reducing greenhouse gas emissions in the atmosphere. In the field crop system, the development of agricultural carbon sequestration projects is important. Emission reductions are achieved by increasing soil organic matter content, promoting soil microbial activity, enhancing soil aggregate stability, improving soil resilience, reducing land erosion, and increasing crop yields. Additionally, agricultural carbon sequestration projects can promote nitrogen cycling in the land, leading to a decrease in nitrogen fertilizer use and nitrous oxide emissions [143]. By optimizing nitrogen application schemes, utilizing organic and biological fertilizers, improving soil nitrogen utilization, and reducing nitrogen loss, nitrous oxide emissions can be lowered [144]. Moreover, these projects can also reduce greenhouse gas emissions by enhancing crop varieties, adjusting planting structures, and implementing multi-season rice cultivation.

## 7. Summary and Prospect

Transitioning from wet paddy fields to dry rice cultivation can substantially decrease methane emissions. Optimizing fertilizer practices, including the type and timing of application, can effectively mitigate nitrous oxide emissions in field crop systems. The implementation of appropriate cultivation techniques and fertilization practices is essential for minimizing greenhouse gas emissions from field crop systems and promoting sustainable agriculture.

Future research could investigate the emission characteristics and influencing factors of greenhouse gases in field crop systems under varying climate conditions. This study investigates the characteristics of greenhouse gas emissions and the factors influencing them under varying climate conditions through detailed experimental studies. It analyzes the impact of different crop types on greenhouse gas emissions within the context of climate change. Furthermore, it aims to develop and implement greenhouse gas reduction technologies in field crop systems, with a focus on the effects of vegetation cover, fertilization techniques, irrigation methods, and agricultural management practices. Additionally, the research examines the use of biochar and other technologies to enhance carbon sequestration and reduce greenhouse gas emissions in field crop systems.

To effectively address the challenges posed by greenhouse gas emissions in field crop systems, it is essential to investigate various national and regional policies aimed at reducing these emissions. By identifying effective strategies and pinpointing policy gaps, we can explore how these initiatives promote the adoption of greenhouse gas reduction measures within the agricultural sector. A comprehensive evaluation of the advantages and disadvantages of different policy approaches will provide valuable insights to policymakers, assisting them in tackling unresolved issues and obstacles in greenhouse gas emission research. By enhancing our understanding of the mechanisms and factors influencing these emissions, we can design and implement effective strategies that not only mitigate greenhouse gases but also advance sustainable agricultural development through ongoing research and dedicated efforts.

## Figures and Tables

**Table 1:** Infrared (IR) and ultraviolet-visible (UV-Vis) spectrometers employ direct absorption techniques to analyze molecules by measuring their absorption of specific wavelengths of electromagnetic radiation, providing information about their functional groups, electronic structure, and molecular transitions.

Item	Content
**IR Spectrometers**	
Principle	Detect the absorption of infrared radiation by molecules, providing information about their functional groups and molecular structure.
Detection	IR radiation is passed through the sample, and the absorption at each wavelength is measured to identify functional groups.
Applications	Identifying organic compounds, determining functional groups, studying molecular structure, environmental monitoring, and medical diagnostics.
**UV-Vis Spectrometers**	
Principle	UV and visible light are absorbed by molecules, causing electron transitions and providing information about their electronic structure.
Detection	Sample is exposed to light of varying wavelengths, and the absorption at each wavelength is measured to determine electronic structure and molecular transitions.
Applications	Quantifying analytes, determining compound purity, identifying unknown substances, clinical diagnostics, environmental monitoring, and food analysis.
**Advantages of direct absorption spectroscopy**	
Direct measurement	Real-time analysis of gas molecule absorption spectra without chemical reactions or labeling
High sensitivity	Can detect very low concentrations of analytes.
Versatility	Can be used for a wide range of gas molecules and applications.
**Limitations of direct absorption spectroscopy**	
Interferences	Other molecules may absorb light at the same wavelength, hindering target analyte detection.
Complexity	The interpretation of absorption spectra can be challenging, especially for complex mixtures.
Size and cost	Spectrometers can be bulky and expensive.

**Table 2:** Denitrification-Decomposition tracked the evolution of CO_2_, N_2_O, and CH_4_ driven by the oxidation-reduction system and matrix concentrations in paddy field soil. Based on the coupled Nernst equation and Michaelis-Menten equation embedded in Denitrification-Decomposition, soil oxidation-reduction system, and dominant oxidizer concentrations were calculated [132].

Dominant Oxidant	Eh (mV)	Reaction	References
Oxygen (O_2_)	650–500	$O_{2}+C=CO_{2}$	[133]
Nitrate (NO_3_^−^) Nitrite (NO_2_^−^) Nitric oxide (NO) Nitrous oxide (N_2_O)	500–200	$NO_{3}^{-}\leftarrow \mathrm{NO}-N$ _2_ $O-NH_{4}^{+}$ $NO_{3}^{-}\to {\mathrm{NO}}_{2}^{-}\to \mathrm{NO}\to N$ _2_ O→N _2_	[134]
Manganese (Mn_4_^+^)	200–100	Mn_4_^+^ + 2e = Mn_2_^+^	[135]
Iron (Fe_3_^+^)	100–0	Fe_3_^+^ + e = Fe_2_^+^	[136]
Sulfate (SO_4_^2−^)	0–−150	SO_4_^2−^ + 10H + 8e = H_2_S + 4H_2_O	[137]
Hydrogen (H_2_)	−150–−300	H_2_ + C → CH_4_ → CO_2_	[138]

Note: $E_{h}=E_{0}+\frac{RT}{nF}\times ln\frac{\left[oxidant\right]}{\left[reductant\right]}$, where $E_{h}$ is redox potential of the oxidation-reduction system (V), $E_{0}$ is standard electromotive force (V), *R* is the gas constant (8.314 J/mol/k), *T* is absolute temperature (273 + t, °C), *n* is transferred electron number, *F* is the Faraday constant (96,485 C/mol), [oxidant] is concentration (mol/L) of dominant oxidant in the system, and [reductant] is concentration (mol/L) of dominant reductant in the system.

## Data Availability

No new data were created or analyzed in this study.

## Supplementary Materials

The following supporting information can be downloaded at: https://www.mdpi.com/article/10.3390/plants13162285/s1, Figure S1: Life cycle assessment: Comprehensive GHG emissions calculations.

## References

[1] Yuan D., Li S., Xu Y.J., Ma S., Zhang K., Le J., Wang Y., Ma B., Jiang P., Zhang L. (2024). Response of Dissolved Carbon Dioxide and Methane Concentration to Warming in Shallow Lakes. Water Res..

[2] Abram N.J., McGregor H.V., Tierney J.E., Evans M.N., McKay N.P., Kaufman D.S. (2016). Early Onset of Industrial-Era Warming across the Oceans and Continents. Nature.

[3] Hayhoe K., Edmonds J., Kopp R., LeGrande A., Sanderson B., Wehner M., Wuebbles D. (2017). Climate Models, Scenarios, and Projections. U.S. Glob. Change Res. Program.

[4] Clarke B., Otto F., Stuart-Smith R., Harrington L. (2022). Extreme weather impacts of climate change: An attribution perspective. Environ. Res. Clim..

[5] Lenton T.M., Xu C., Abrams J.F., Ghadiali A., Loriani S., Sakschewski B., Zimm C., Ebi K.L., Dunn R.R., Svenning J.-C. (2023). Quantifying the human cost of global warming. Nat. Sustain..

[6] Jones M.W., Peters G.P., Gasser T., Andrew R.M., Schwingshackl C., Gütschow J., Houghton R.A., Friedlingstein P., Pongratz J., Le Quéré C. (2023). National Contributions to Climate Change Due to Historical Emissions of Carbon Dioxide, Methane, and Nitrous Oxide since 1850. Sci. Data.

[7] Basheer S., Wang X., Farooque A.A., Nawaz R.A., Pang T., Neokye E.O. (2024). A Review of Greenhouse Gas Emissions from Agricultural Soil. Sustainability.

[8] Altieri M.A., Nicholls C.I., Montalba R. (2017). Technological Approaches to Sustainable Agriculture at a Crossroads: An Agroecological Perspective. Sustainability.

[9] Durham T.C., Mizik T. (2021). Comparative Economics of Conventional, Organic, and Alternative Agricultural Production Systems. Economies.

[10] Sharma G., Shrestha S., Kunwar S., Tseng T.M. (2021). Crop Diversification for Improved Weed Management: A Review. Agriculture.

[11] Wang X., Wang G., Turner N.C., Xing Y., Li M., Guo T. (2020). Determining optimal mulching, planting density, and nitrogen application to increase maize grain yield and nitrogen translocation efficiency in Northwest China. BMC Plant Biol..

[12] Xing Y., Zhang T., Jiang W., Li P., Shi P., Xu G., Cheng S., Cheng Y., Fan Z., Wang X. (2022). Effects of Irrigation and Fertilization on Different Potato Varieties Growth, Yield and Resources Use Efficiency in the Northwest China. Agric. Water Manag..

[13] Sukhoveeva O., Karelin D., Lebedeva T., Pochikalov A., Ryzhkov O., Suvorov G., Zolotukhin A. (2023). Greenhouse gases fluxes and carbon cycle in agroecosystems under humid continental climate conditions. Agric. Ecosyst. Environ..

[14] Zheng J., Scheibe T.D., Boye K., Song H.-S. (2024). Thermodynamic control on the decomposition of organic matter across different electron acceptors. Soil Biol. Biochem..

[15] Zhang H., Liang Q., Peng Z., Zhao Y., Tan Y., Zhang X., Bol R. (2023). Response of Greenhouse Gases Emissions and Yields to Irrigation and Straw Practices in Wheat-Maize Cropping System. Agric. Water Manag..

[16] Bai J., Song J., Chen D., Zhang Z., Yu Q., Ren G., Han X., Wang X., Ren C., Yang G. (2023). Biochar combined with N fertilization and straw return in wheat-maize agroecosystem: Key practices to enhance crop yields and minimize carbon and nitrogen footprints. Agric. Ecosyst. Environ..

[17] Qian H., Zhu X., Huang S., Linquist B., Kuzyakov Y., Wassmann R., Minamikawa K., Martinez-Eixarch M., Yan X., Zhou F. (2023). Greenhouse gas emissions and mitigation in rice agriculture. Nat. Rev. Earth Environ..

[18] Tian S., Xu Y., Wang Q., Zhang Y., Yuan X., Ma Q., Feng X., Ma H., Liu J., Liu C. (2023). The Effect of Optimizing Chemical Fertilizers Consumption Structure to Promote Environmental Protection, Crop Yield and Reduce Greenhouse Gases Emission in China. Sci. Total Environ..

[19] Nunes L. (2023). The Rising Threat of Atmospheric CO_2_: A Review on the Causes, Impacts, and Mitigation Strategies. Environments.

[20] Thakur S., Solanki H. (2022). Role of Methane in Climate Change and Options for Mitigation—A Brief Review. Int. Assoc. Biol. Comput. Dig..

[21] Walling E., Vaneeckhaute C. (2020). Greenhouse gas emissions from inorganic and organic fertilizer production and use: A review of emission factors and their variability. J. Environ. Manag..

[22] Fuchs A., Dalby F.R., Liu D., Kai P., Feilberg A. (2021). Improved effect of manure acidification technology for gas emission mitigation by substituting sulfuric acid with acetic acid. Clean. Eng. Technol..

[23] Yu K., Fang X., Zhang Y., Miao Y., Liu S., Zou J. (2021). Low greenhouse gases emissions associated with high nitrogen use efficiency under optimized fertilization regimes in double-rice cropping systems. Appl. Soil Ecol..

[24] Sanz-Cobena A., Lassaletta L., Aguilera E., del Prado A., Garnier J., Billen G., Iglesias A., Sánchez B., Guardia G., Abalos D. (2017). Strategies for greenhouse gas emissions mitigation in Mediterranean agriculture: A review. Agric. Ecosyst. Environ..

[25] Mehmood T., Hassan M.A., Li X., Ashraf A., Rehman S., Bilal M., Obodo R.M., Mustafa B., Shaz M., Bibi S. (2022). Mechanism Behind Sources and Sinks of Major Anthropogenic Greenhouse Gases. Climate Change Alleviation for Sustainable Progression.

[26] Waha K., Dietrich J.P., Portmann F.T., Siebert S., Thornton P.K., Bondeau A., Herrero M. (2020). Multiple cropping systems of the world and the potential for increasing cropping intensity. Glob. Environ. Chang..

[27] Kweku D.W., Bismark O., Maxwell A., Desmond K.A., Danso K.B., Oti-Mensah E.A., Quachie A.T., Adormaa B.B. (2018). Greenhouse Effect: Greenhouse Gases and Their Impact on Global Warming. J. Sci. Res. Rep..

[28] Sakadevan K., Nguyen M.L. (2017). Livestock Production and Its Impact on Nutrient Pollution and Greenhouse Gas Emissions. Adv. Agron..

[29] Alkharabsheh H.M., Seleiman M.F., Battaglia M.L., Shami A., Jalal R.S., Alhammad B.A., Almutairi K.F., Al-Saif A.M. (2021). Biochar and Its Broad Impacts in Soil Quality and Fertility, Nutrient Leaching and Crop Productivity: A Review. Agronomy.

[30] Raza A., Razzaq A., Mehmood S.S., Zou X., Zhang X., Lv Y., Xu J. (2019). Impact of Climate Change on Crops Adaptation and Strategies to Tackle Its Outcome: A Review. Plants.

[31] Lesk C., Rowhani P., Ramankutty N. (2016). Influence of Extreme Weather Disasters on Global Crop Production. Nature.

[32] Chaudhary S., Devi P., HanumanthaRao B., Jha U.C., Sharma K.D., Prasad P.V., Kumar S., Siddique K.H.M., Nayyar H. (2022). Physiological and Molecular Approaches for Developing Thermotolerance in Vegetable Crops: A Growth, Yield and Sustenance Perspective. Front. Plant Sci..

[33] Rani V., Bhatia A., Kaushik R. (2021). Inoculation of plant growth promoting-methane utilizing bacteria in different N-fertilizer regime influences methane emission and crop growth of flooded paddy. Sci. Total. Environ..

[34] Chataut G., Bhatta B., Joshi D., Subedi K., Kafle K. (2023). Greenhouse Gases Emission from Agricultural Soil: A Review. J. Agric. Food Res..

[35] Sun S., Gu X., Zhang M., Tang L., He S., Huang J. (2021). Biological iron nitrogen cycle in ecological floating bed: Nitrogen removal improvement and nitrous oxide emission reduction. Environ. Pollut..

[36] Moufid M., Bouchikhi B., Tiebe C., Bartholmai M., El Bari N. (2021). Assessment of Outdoor Odor Emissions from Polluted Sites Using Simultaneous Thermal Desorption-Gas Chromatography-Mass Spectrometry (Td-Gc-Ms), Electronic Nose in Conjunction with Advanced Multivariate Statistical Approaches. Atmos. Environ..

[37] Li C., Mosier A., Wassmann R., Cai Z., Zheng X., Huang Y., Tsuruta H., Boonjawat J., Lantin R. (2004). Modeling greenhouse gas emissions from rice-based production systems: Sensitivity and upscaling. Glob. Biogeochem. Cycles.

[38] Simonenko N.P., Fisenko N.A., Fedorov F.S., Simonenko T.L., Mokrushin A.S., Simonenko E.P., Korotcenkov G., Sysoev V.V., Sevastyanov V.G., Kuznetsov N.T. (2022). Printing Technologies as an Emerging Approach in Gas Sensors: Survey of Literature. Sensors.

[39] Hergoualc’h K., Mueller N., Bernoux M., Kasimir Ä., van der Weerden T.J., Ogle S.M. (2021). Improved accuracy and reduced uncertainty in greenhouse gas inventories by refining the IPCC emission factor for direct N_2_O emissions from nitrogen inputs to managed soils. Glob. Chang. Biol..

[40] Stetter C., Sauer J. (2022). Greenhouse Gas Emissions and Eco-Performance at Farm Level: A Parametric Approach. Environ. Resour. Econ..

[41] Guo C., Liu X., He X. (2022). A global meta-analysis of crop yield and agricultural greenhouse gas emissions under nitrogen fertilizer application. Sci. Total. Environ..

[42] Mouronte-López M.L., Subirán M. (2023). Analysis of Worldwide Greenhouse and Carbon Monoxide Gas Emissions: Which Countries Exhibit a Special Pattern? A Closer Look Via Twitter. Int. J. Environ. Res..

[43] Li J., Tian Y., Zhang Y., Xie K. (2021). Spatializing environmental footprint by integrating geographic information system into life cycle assessment: A review and practice recommendations. J. Clean. Prod..

[44] Hejazi M.I., Edmonds J., Clarke L., Kyle P., Davies E., Chaturvedi V., Wise M., Patel P., Eom J., Calvin K. (2014). Integrated assessment of global water scarcity over the 21st century under multiple climate change mitigation policies. Hydrol. Earth Syst. Sci..

[45] De Feo G., Ferrara C., Iuliano C., Grosso A. (2016). LCA of the Collection, Transportation, Treatment and Disposal of Source Separated Municipal Waste: A Southern Italy Case Study. Sustainability.

[46] Hannouf M., Assefa G. (2018). A Life Cycle Sustainability Assessment-Based Decision-Analysis Framework. Sustainability.

[47] Miliute-Plepiene J., Sundqvist J.O. (2024). Assessing the Potential Climate Impacts and Benefits of Waste Prevention and Management: A Case Study of Sweden. Sustainability.

[48] Cheng K., Ogle S.M., Parton W.J., Pan G. (2013). Simulating greenhouse gas mitigation potentials for Chinese Croplands using the DAYCENT ecosystem model. Glob. Chang. Biol..

[49] Cerqueira V., Torgo L., Mozetič I. (2020). Evaluating Time Series Forecasting Models: An Empirical Study on Performance Estimation Methods. Mach. Learn..

[50] Wu G., Chen X.-M., Ling J., Li F., Li F.-Y., Peixoto L., Wen Y., Zhou S.-L. (2020). Effects of soil warming and increased precipitation on greenhouse gas fluxes in spring maize seasons in the North China Plain. Sci. Total. Environ..

[51] Griffis T.J., Chen Z., Baker J.M., Wood J.D., Millet D.B., Lee X., Venterea R.T., Turner P.A. (2017). Nitrous oxide emissions are enhanced in a warmer and wetter world. Proc. Natl. Acad. Sci. USA.

[52] Martínez-Eixarch M., Alcaraz C., Viñas M., Noguerol J., Aranda X., Prenafeta-Boldú F.-X., Català-Forner M., Fennessy M.S., Ibáñez C. (2021). The main drivers of methane emissions differ in the growing and flooded fallow seasons in Mediterranean rice fields. Plant Soil.

[53] Li J., Wang S., Shi Y., Zhang L., Wu Z. (2021). Do Fallow Season Cover Crops Increase N_2_O or CH_4_ Emission from Paddy Soils in the Mono-Rice Cropping System?. Agronomy.

[54] Lim J., Wehmeyer H., Heffner T., Aeppli M., Gu W., Kim P.J., Horn M., Ho A. (2024). Resilience of Aerobic Methanotrophs in Soils; Spotlight on the Methane Sink under Agriculture. FEMS Microbiol. Ecol..

[55] Dimitriou K., Bougiatioti A., Ramonet M., Pierros F., Michalopoulos P., Liakakou E., Solomos S., Quehe P.-Y., Delmotte M., Gerasopoulos E. (2021). Greenhouse Gases (CO_2_ and CH_4_) at an Urban Background Site in Athens, Greece: Levels, Sources and Impact of Atmospheric Circulation. Atmos. Environ..

[56] Lan R., Eastham S.D., Liu T., Norford L.K., Barrett S.R.H. (2022). Air quality impacts of crop residue burning in India and mitigation alternatives. Nat. Commun..

[57] Ainsworth E.A., Lemonnier P., Wedow J.M. (2019). The influence of rising tropospheric carbon dioxide and ozone on plant productivity. Plant Biol..

[58] Song T., Liu Y., Kolton M., Wilson R.M., Keller J.K., Rolando J.L., Chanton J.P., Kostka J.E. (2023). Porewater Constituents Inhibit Microbially Mediated Greenhouse Gas Production (Ghg) and Regulate the Response of Soil Organic Matter Decomposition to Warming in Anoxic Peat from a Sphagnum-Dominated Bog. FEMS Microbiol. Ecol..

[59] Shaaban M. (2024). Microbial pathways of nitrous oxide emissions and mitigation approaches in drylands. J. Environ. Manag..

[60] Li X., Petersen S.O., Sørensen P., Olesen J.E. (2015). Effects of contrasting catch crops on nitrogen availability and nitrous oxide emissions in an organic cropping system. Agric. Ecosyst. Environ..

[61] Dusenge M.E., Duarte A.G., Way D.A. (2019). Plant Carbon Metabolism and Climate Change: Elevated CO_2_ and Temperature Impacts on Photosynthesis, Photorespiration and Respiration. New Phytol..

[62] Shah I.H., Manzoor M.A., Jinhui W., Li X., Hameed M.K., Rehaman A., Li P., Zhang Y., Niu Q., Chang L. (2024). Comprehensive review: Effects of climate change and greenhouse gases emission relevance to environmental stress on horticultural crops and management. J. Environ. Manag..

[63] Ghimire R., Norton U., Bista P., Obour A.K., Norton J.B. (2016). Soil organic matter, greenhouse gases and net global warming potential of irrigated conventional, reduced-tillage and organic cropping systems. Nutr. Cycl. Agroecosyst..

[64] Maraseni T.N., Deo R.C., Qu J., Gentle P., Neupane P.R. (2018). An international comparison of rice consumption behaviours and greenhouse gas emissions from rice production. J. Clean. Prod..

[65] Thilakarathna S.K., Hernandez-Ramirez G., Puurveen D., Kryzanowski L., Lohstraeter G., Powers L.A., Quan N., Tenuta M. (2020). Nitrous Oxide Emissions and Nitrogen Use Efficiency in Wheat: Nitrogen Fertilization Timing and Formulation, Soil Nitrogen, and Weather Effects. Soil Sci. Soc. Am. J..

[66] Liu X., Peñuelas J., Sardans J., Fang Y., Wiesmeier M., Wu L., Chen X., Chen Y., Jin Q., Wang W. (2021). Response of soil nutrient concentrations and stoichiometry, and greenhouse gas carbon emissions linked to change in land-use of paddy fields in China. Catena.

[67] Mallareddy M., Thirumalaikumar R., Balasubramanian P., Naseeruddin R., Nithya N., Mariadoss A., Eazhilkrishna N., Choudhary A.K., Deiveegan M., Subramanian E. (2023). Maximizing Water Use Efficiency in Rice Farming: A Comprehensive Review of Innovative Irrigation Management Technologies. Water.

[68] Rosenheim J.A., Cass B.N., Kahl H., Steinmann K.P. (2020). Variation in pesticide use across crops in California agriculture: Economic and ecological drivers. Sci. Total. Environ..

[69] Kumar R., Karmakar S., Minz A., Singh J., Kumar A., Kumar A. (2021). Assessment of Greenhouse Gases Emission in Maize-Wheat Cropping System Under Varied N Fertilizer Application Using Cool Farm Tool. Front. Environ. Sci..

[70] Yun J., Wang C., Zhang F., Chen L., Sun Z., Cai Y., Luo Y., Liao J., Wang Y., Cha Y. (2023). A nitrogen fixing symbiosis-specific pathway required for legume flowering. Sci. Adv..

[71] Korres N.E., Singh A., Prasad S. (2023). Agricultural Residues Management: Life Cycle Assessment Implications for Sustainable Agricultural Practices and Reduction of Greenhouse Gases Emissions. Adv. Agron..

[72] Miglani G.S., Kaur R., Sharma P., Gupta N. (2020). Leveraging photosynthetic efficiency toward improving crop yields. J. Crop. Improv..

[73] Grzebisz W., Zielewicz W., Przygocka-Cyna K. (2022). Deficiencies of Secondary Nutrients in Crop Plants—A Real Challenge to Improve Nitrogen Management. Agronomy.

[74] Elnahal A.S., Abdo A.I., Desoky E.-S.M., Selem E., Rady M.M. (2022). Traditional, Modern, and Molecular Strategies for Improving the Efficiency of Nitrogen Use in Crops for Sustainable Agriculture: A Fresh Look at an Old Issue. J. Soil Sci. Plant Nutr..

[75] Goldan E., Nedeff V., Barsan N., Culea M., Panainte-Lehadus M., Mosnegutu E., Tomozei C., Chitimus D., Irimia O. (2023). Assessment of Manure Compost Used as Soil Amendment—A Review. Processes.

[76] Lyu H., Li Y., Wang Y., Wang P., Shang Y., Yang X., Wang F., Yu A. (2024). Drive Soil Nitrogen Transformation and Improve Crop Nitrogen Absorption and Utilization-a Review of Green Manure Applications. Front. Plant Sci..

[77] Li H., Mei X., Wang J., Huang F., Hao W., Li B. (2020). Drip fertigation significantly increased crop yield, water productivity and nitrogen use efficiency with respect to traditional irrigation and fertilization practices: A meta-analysis in China. Agric. Water Manag..

[78] Amirahmadi E., Ghorbani M., Moudrý J., Bernas J., Mukosha C.E., Hoang T.N. (2024). Environmental Assessment of Dryland and Irrigated Winter Wheat Cultivation under Compost Fertilization Strategies. Plants.

[79] Yu Y., Jiao Y., Yang W., Song C., Zhang J., Liu Y. (2022). Mechanisms underlying nitrous oxide emissions and nitrogen leaching from potato fields under drip irrigation and furrow irrigation. Agric. Water Manag..

[80] Slamini M., Sbaa M., Arabi M., Darmous A. (2022). Review on Partial Root-Zone Drying Irrigation: Impact on Crop Yield, Soil and Water Pollution. Agric. Water Manag..

[81] Bwambale E., Abagale F.K., Anornu G.K. (2022). Smart Irrigation Monitoring and Control Strategies for Improving Water Use Efficiency in Precision Agriculture: A Review. Agric. Water Manag..

[82] Patra K., Parihar C.M., Nayak H.S., Rana B., Singh V.K., Jat S.L., Panwar S., Parihar M.D., Singh L.K., Sidhu H.S. (2021). Water budgeting in conservation agriculture-based sub-surface drip irrigation in tropical maize using HYDRUS-2D in South Asia. Sci. Rep..

[83] Hu Y., Li D., Wu Y., Liu S., Li L., Chen W., Wu S., Meng Q., Feng H., Siddique K.H. (2023). Mitigating greenhouse gas emissions by replacing inorganic fertilizer with organic fertilizer in wheat–maize rotation systems in China. J. Environ. Manag..

[84] Tyagi J., Ahmad S., Malik M. (2022). Nitrogenous Fertilizers: Impact on Environment Sustainability, Mitigation Strategies, and Challenges. Int. J. Environ. Sci. Technol..

[85] Abalos D., Recous S., Butterbach-Bahl K., De Notaris C., Rittl T.F., Topp C.F., Petersen S.O., Hansen S., Bleken M.A., Rees R.M. (2022). A review and meta-analysis of mitigation measures for nitrous oxide emissions from crop residues. Sci. Total. Environ..

[86] Schmatz R., Recous S., Weiler D.A., Pilecco G.E., Schu A.L., Giovelli R.L., Giacomini S.J. (2020). How the mass and quality of wheat and vetch mulches affect drivers of soil N_2_O emissions. Geoderma.

[87] Or D., Keller T., Schlesinger W.H. (2021). Natural and Managed Soil Structure: On the Fragile Scaffolding for Soil Functioning. Soil Tillage Res..

[88] Naorem A., Jayaraman S., Sinha N.K., Mohanty M., Chaudhary R.S., Hati K.M., Mandal A., Thakur J.K., Patra A.K., Srinivasarao C. (2023). Eight-Year Impacts of Conservation Agriculture on Soil Quality, Carbon Storage, and Carbon Emission Footprint. Soil Tillage Res..

[89] Raza T., Qadir M.F., Khan K.S., Eash N.S., Yousuf M., Chatterjee S., Manzoor R., ur Rehman S., Oetting J.N. (2023). Unrevealing the potential of microbes in decomposition of organic matter and release of carbon in the ecosystem. J. Environ. Manag..

[90] Menegat S., Ledo A., Tirado R. (2022). Greenhouse gas emissions from global production and use of nitrogen synthetic fertilisers in agriculture. Sci. Rep..

[91] Eskander S.M., Fankhauser S. (2020). Reduction in greenhouse gas emissions from national climate legislation. Nat. Clim. Chang..

[92] Muhammad I., Lv J.Z., Wang J., Ahmad S., Farooq S., Ali S., Zhou X.B. (2022). Regulation of soil microbial community structure and biomass to mitigate soil greenhouse gas emission. Front. Microbiol..

[93] Moore C.E., Meacham-Hensold K., Lemonnier P., Slattery R.A., Benjamin C., Bernacchi C.J., Lawson T., Cavanagh A.P. (2021). The effect of increasing temperature on crop photosynthesis: From enzymes to ecosystems. J. Exp. Bot..

[94] Zhang H., Yao X., Zeng W., Fang Y., Wang W. (2020). Depth dependence of temperature sensitivity of soil carbon dioxide, nitrous oxide and methane emissions. Soil Biol. Biochem..

[95] Ramzan S., Rasool T., Bhat R.A., Ahmad P., Ashraf I., Rashid N., ul Shafiq M., Mir I.A. (2020). Agricultural soils a trigger to nitrous oxide: A persuasive greenhouse gas and its management. Environ. Monit. Assess..

[96] Long S.P., Taylor S.H., Burgess S.J., Carmo-Silva E., Lawson T., De Souza A.P., Leonelli L., Wang Y. (2022). Into the shadows and back into sunlight: Photosynthesis in fluctuating light. Annu. Rev. Plant Biol..

[97] Yu H., Zhang Z., Zhang Y., Song Q., Fan P., Xi B., Tan W. (2021). Effects of microplastics on soil organic carbon and greenhouse gas emissions in the context of straw incorporation: A comparison with different types of soil. Environ. Pollut..

[98] Ferdush J., Paul V. (2021). A review on the possible factors influencing soil inorganic carbon under elevated CO_2_. Catena.

[99] Bussell J., Crotty F., Stoate C. (2021). Comparison of compaction alleviation methods on soil health and greenhouse gas emissions. Land.

[100] Ren X., Tang J., Liu X., Liu Q. (2020). Effects of microplastics on greenhouse gas emissions and the microbial community in fertilized soil. Environ. Pollut..

[101] Wu H., Cui H., Fu C., Li R., Qi F., Liu Z., Yang G., Xiao K., Qiao M. (2023). Unveiling the crucial role of soil microorganisms in carbon cycling: A review. Sci. Total Environ..

[102] Katzin D., Van Henten E.J., Van Mourik S. (2022). Process-based greenhouse climate models: Genealogy, current status, and future directions. Agric. Syst..

[103] Xu K., Guo X., He J., Yu B., Tan J., Guo Y. (2022). A study on temperature spatial distribution of a greenhouse under solar load with considering crop transpiration and optical effects. Energy Convers. Manag..

[104] Zeraatpisheh M., Garosi Y., Owliaie H.R., Ayoubi S., Taghizadeh-Mehrjardi R., Scholten T., Xu M. (2022). Improving the spatial prediction of soil organic carbon using environmental covariates selection: A comparison of a group of environmental covariates. Catena.

[105] da Rosa Ferraz Jardim A.M., de Morais J.E.F., de Souza L.S.B., da Silva T.G.F. (2022). Understanding interactive processes: A review of CO_2_ flux, evapotranspiration, and energy partitioning under stressful conditions in dry forest and agricultural environments. Environ. Monit. Assess..

[106] Hamrani A., Akbarzadeh A., Madramootoo C.A. (2020). Machine learning for predicting greenhouse gas emissions from agricultural soils. Sci. Total Environ..

[107] Sherwood S.C., Webb M.J., Annan J.D., Armour K.C., Forster P.M., Hargreaves J.C., Hegerl G., Klein S.A., Marvel K.D., Rohling E.J. (2020). An assessment of Earth’s climate sensitivity using multiple lines of evidence. Rev. Geophys..

[108] Schauberger B., Jägermeyr J., Gornott C. (2020). A systematic review of local to regional yield forecasting approaches and frequently used data resources. Eur. J. Agron..

[109] Yin R., Wang D., Zhao S., Lou Z., Shen G. (2021). Wearable sensors-enabled human–machine interaction systems: From design to application. Adv. Funct. Mater..

[110] Ma Z., Dey S., Christopher S., Liu R., Bi J., Balyan P., Liu Y. (2022). A review of statistical methods used for developing large-scale and long-term PM_2.5_ models from satellite data. Remote Sens. Environ..

[111] Zhan S., Chong A. (2021). Data requirements and performance evaluation of model predictive control in buildings: A modeling perspective. Renew. Sustain. Energy Rev..

[112] Hou D., Hassan I., Wang L. (2021). Review on building energy model calibration by Bayesian inference. Renew. Sustain. Energy Rev..

[113] Tang Y., Luan X., Sun J., Zhao J., Yin Y., Wang Y., Sun S. (2021). Impact assessment of climate change and human activities on GHG emissions and agricultural water use. Agric. For. Meteorol..

[114] Bhattacharyya S.S., Leite F.F.G.D., France C.L., Adekoya A.O., Ros G.H., de Vries W., Melchor-Martínez E.M., Iqbal H.M., Parra-Saldívar R. (2022). Soil carbon sequestration, greenhouse gas emissions, and water pollution under different tillage practices. Sci. Total Environ..

[115] Wang C., Amon B., Schulz K., Mehdi B. (2021). Factors that influence nitrous oxide emissions from agricultural soils as well as their representation in simulation models: A review. Agronomy.

[116] Gualtieri G. (2022). Analysing the uncertainties of reanalysis data used for wind resource assessment: A critical review. Renew. Sustain. Energy Rev..

[117] Pianosi F., Beven K., Freer J., Hall J.W., Rougier J., Stephenson D.B., Wagener T. (2016). Sensitivity analysis of environmental models: A systematic review with practical workflow. Environ. Model. Softw..

[118] Tiedeman C.R., Green C.T. (2013). Effect of correlated observation error on parameters, predictions, and uncertainty. Water Resour. Res..

[119] Omia E., Bae H., Park E., Kim M.S., Baek I., Kabenge I., Cho B.-K. (2023). Remote sensing in field crop monitoring: A comprehensive review of sensor systems, data analyses and recent advances. Remote Sens..

[120] Yin W., Gou Z., Fan Z., Hu F., Fan H., Zhao C., Yu A., Chai Q. (2022). No-tillage with straw mulching and re-using old film boost crop yields and mitigate soil N_2_O emissions in wheat-maize intercropping at arid irrigated regions. Field Crops Res..

[121] Islam M.U., Jiang F., Guo Z., Liu S., Peng X. (2023). Impacts of straw return coupled with tillage practices on soil organic carbon stock in upland wheat and maize croplands in China: A meta-analysis. Soil Tillage Res..

[122] Wang X., Jia Z., Liang L., Zhao Y., Yang B., Ding R., Wang J., Nie J. (2018). Changes in soil characteristics and maize yield under straw returning system in dryland farming. Field Crops Res..

[123] Glaze-Corcoran S., Hashemi M., Sadeghpour A., Jahanzad E., Afshar R.K., Liu X., Herbert S.J. (2020). Understanding intercropping to improve agricultural resiliency and environmental sustainability. Adv. Agron..

[124] Rastogi M., Verma S., Kumar S., Bharti S., Kumar G., Azam K., Singh V. (2023). Soil health and sustainability in the age of organic amendments: A review. Int. J. Environ. Clim. Change.

[125] Ibrahim M.M., Zhang H., Guo L., Chen Y., Heiling M., Zhou B., Mao Y. (2021). Biochar interaction with chemical fertilizer regulates soil organic carbon mineralization and the abundance of key C-cycling-related bacteria in rhizosphere soil. Eur. J. Soil Biol..

[126] Shah K.K., Modi B., Pandey H.P., Subedi A., Aryal G., Pandey M., Shrestha J. (2021). Diversified crop rotation: An approach for sustainable agriculture production. Adv. Agric..

[127] Wang X., Chen Y., Yang K., Duan F., Liu P., Wang Z., Wang J. (2021). Effects of legume intercropping and nitrogen input on net greenhouse gas balances, intensity, carbon footprint and crop productivity in sweet maize cropland in South China. J. Clean. Prod..

[128] Cha-un N., Chidthaisong A., Yagi K., Sudo S., Towprayoon S. (2017). Greenhouse gas emissions, soil carbon sequestration and crop yields in a rain-fed rice field with crop rotation management. Agric. Ecosyst. Environ..

[129] Yang T., Siddique K.H., Liu K. (2020). Cropping systems in agriculture and their impact on soil health-A review. Glob. Ecol. Conserv..

[130] Stephan A., Stephan L. (2016). Life cycle energy and cost analysis of embodied, operational and user-transport energy reduction measures for residential buildings. Appl. Energy.

[131] Kumar S., Sindhu S.S., Kumar R. (2022). Biofertilizers: An ecofriendly technology for nutrient recycling and environmental sustainability. Curr. Res. Microb. Sci..

[132] Ravindra K., Singh T., Mor S. (2019). Emissions of air pollutants from primary crop residue burning in India and their mitigation strategies for cleaner emissions. J. Clean. Prod..

[133] Rohe L., Apelt B., Vogel H.-J., Well R., Wu G.-M., Schlüter S. (2021). Denitrification in soil as a function of oxygen availability at the microscale. Biogeosciences.

[134] Zhang F., Li P., Chen M., Wu J., Zhu N., Wu P., Chiang P., Hu Z. (2015). Effect of operational modes on nitrogen removal and nitrous oxide emission in the process of simultaneous nitrification and denitrification. Chem. Eng. J..

[135] Ren Z., Zhang H., Wang G., Pan Y., Yu Z., Long H. (2020). Effect of Calcination Temperature on the Activation Performance and Reaction Mechanism of Ce–Mn–Ru/TiO_2_ Catalysts for Selective Catalytic Reduction of NO with NH_3_. ACS Omega.

[136] Cisternas J., Rodríguez C., Serrano J., Leiva E. (2023). Study of the key biotic and abiotic parameters influencing ammonium removal from wastewaters by Fe^3+^-mediated anaerobic ammonium oxidation (Feammox). Chemosphere.

[137] Ai T., Zou L., Cheng H., Luo Z., Al-Rekabi W.S., Li H., Fu Q., He Q., Ai H. (2022). The potential of electrotrophic denitrification coupled with sulfur recycle in MFC and its responses to COD/SO_4_^2−^ ratios. Chemosphere.

[138] Han Y., Yang P., Feng Y., Wang N., Yuan X., An J., Liu J., Li N., He W. (2023). Liquid-gas phase transition enables microbial electrolysis and H2-based membrane biofilm hybrid system to degrade organic pollution and achieve effective hydrogenotrophic denitrification of groundwater. Chemosphere.

[139] Villoria-Sáez P., Tam V.W., del Río Merino M., Arrebola C.V., Wang X. (2016). Effectiveness of greenhouse-gas Emission Trading Schemes implementation: A review on legislations. J. Clean. Prod..

[140] Wei Y., Liang X., Xu L., Kou G., Chevallier J. (2023). Trading, storage, or penalty? Uncovering firms’ decision-making behavior in the Shanghai emissions trading scheme: Insights from agent-based modeling. Energy Econ..

[141] Wang M., Zhou P. (2022). A two-step auction-refund allocation rule of CO_2_ emission permits. Energy Econ..

[142] Karsenty A., Vogel A., Castell F. (2014). “Carbon rights”, REDD+ and payments for environmental services. Environ. Sci. Policy.

[143] Guenet B., Gabrielle B., Chenu C., Arrouays D., Balesdent J., Bernoux M., Bruni E., Caliman J.P., Cardinael R., Chen S. (2021). Can N_2_O emissions offset the benefits from soil organic carbon storage?. Glob. Change Biol..

[144] Pan S.-Y., He K.-H., Lin K.-T., Fan C., Chang C.-T. (2022). Addressing nitrogenous gases from croplands toward low-emission agriculture. Npj Clim. Atmos. Sci..

