# Supplementary material for: Impact of Agricultural Activities on Climate Change: A Review of Greenhouse Gas Emission Patterns in Field Crop Systems
Source: Plants (Basel). 2024 Aug 17;13(16):2285. doi: 10.3390/plants13162285 (PMC11360188; doi:10.3390/plants13162285)
Supplement: Supplementary file 1 [file plants-13-02285-s001.zip › plants-3153776-supplementary.pdf]

Manuscript title:

Impact of Agricultural Activities on Climate Change: A Review of Greenhouse Gas Emission Patterns in Field Crop Systems

Manuscript type:

Review Article

Author list:

Yingying Xing, Xiukang Wang*

Affiliations of authors:

Key Laboratory of Applied Ecology of Loess Plateau, College of Life Science, Yan'an University, Yan'an, Shaanxi 716000, China

Corresponding author:

Key Laboratory of Applied Ecology of Loess Plateau, College of Life Science,

Yan'an University, Yan'an, Shaanxi 716000, China

wangxiukang@126.com (Xiukang Wang)

Telephone and fax: +86 911 2332030

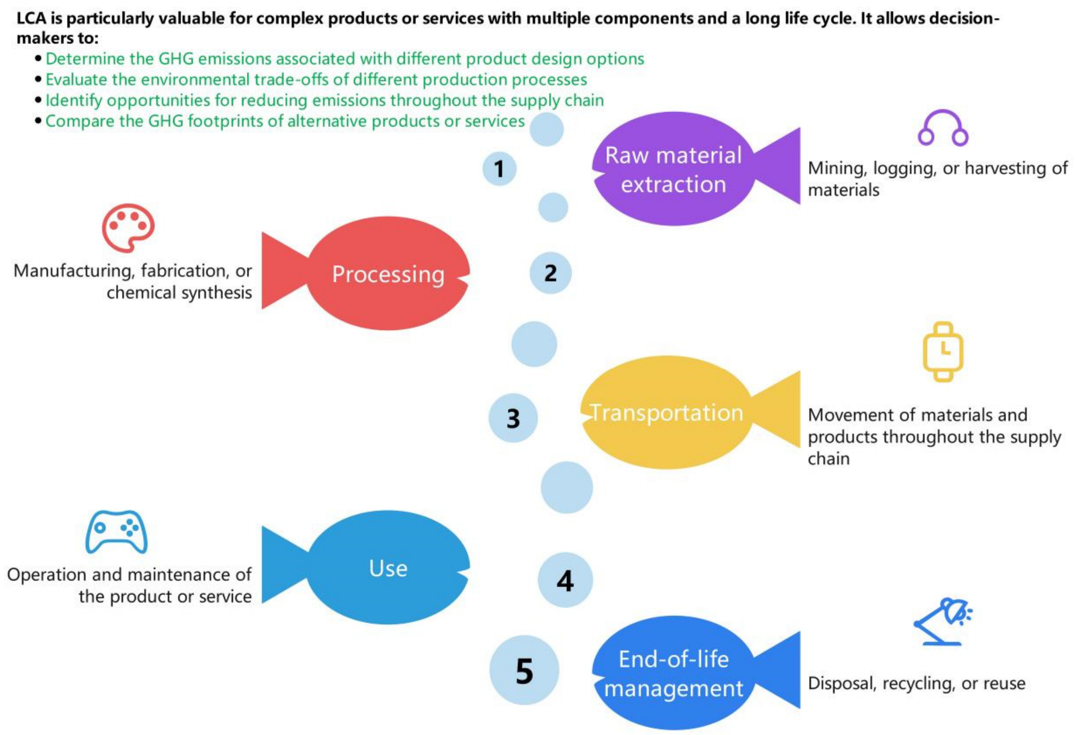

Figure S1. Life cycle assessment: Comprehensive GHG emissions calculations.
